# Chronic Pain Modulates Empathic Responses in People with Spinal Cord Injury

**DOI:** 10.3390/jcm14165878

**Published:** 2025-08-20

**Authors:** Giulia Galli, Luca Sebastianelli, Giorgia De Santis, Giorgio Scivoletto, Marta Mascanzoni, Mariella Pazzaglia

**Affiliations:** 1 Body and Action Lab and Spinal Center and Spinal Rehabilitation Laboratory, IRCCS Fondazione Santa Lucia, 00179 Rome, Italy; g.galli@hsantalucia.it (G.G.); luca.sebastianelli@uniroma1.it (L.S.); giorgia.desantis@uniroma1.it (G.D.S.); g.scivoletto@hsantalucia.it (G.S.); 2 Department of Psychology, Sapienza University of Rome, 00185 Rome, Italy; 3 Department of Human Sciences, LUMSA University of Rome, 00193 Roma, Italy; m.mascanzoni@lumsa.it

**Keywords:** empathy, pain, spinal cord injury, embodied cognition, rehabilitation

## Abstract

**Background/Objectives:** While the correlation between bodily states and cognitive processing has been extensively investigated concerning pain elaboration, little is known about how chronic, subjectively experienced pain (self-pain) following traumatic spinal cord injury (SCI) affects embodied cognition, such as empathy for pain. This study aimed to determine whether individuals with SCI differ from healthy controls in these cognitive responses, and if such differences can be quantified through varying reaction times to pain-related and non-pain-related stimuli involving others. **Methods:** We assessed reactions to others’ pain through behavioral responses in a classification task involving 15 participants with SCI (13 men; age range 19–56 years) and 15 healthy controls (11 men; age range 25–48 years). Additionally, we measured general empathic dispositions using the Interpersonal Reactivity Index (IRI) and subjective pain intensity using a numeric rating scale following standard guidelines for neuropathic pain assessment. **Results:** The findings revealed that participants with SCI exhibited lower empathy levels (IRI: mean SCI = 55.06 ± 3.64) than healthy controls (IRI: mean HC = 67.6 ± 2.46), as measured through both cognitive and affective components. We found that higher chronic pain unpleasantness was associated with lower empathic dispositions (r = −0.63; *p* = 0.01) in participants with SCI. Compared to healthy controls, individuals with SCI exhibited a reduced empathic response when observing others in pain from a third-person perspective. **Conclusions:** These findings reveal an association between chronic pain following SCI and diminished empathic processing, offering new insights into the mechanisms underlying interpersonal reactivity after SCI.

## 1. Introduction

Empathy, often seen as a multidimensional process, helps us understand others [1], interact socially [2], and encourage helpful actions [3]. This complex process encompasses a range of components, from automatic and involuntary responses elicited by observing others to more deliberate, cognitively driven mechanisms [4]. Cognitive empathy is the ability to understand another’s feelings and closely related to theory of mind, such as perspective taking [5]. Affective empathy refers to the ability to recognize and vicariously share the emotions of others such as sensorimotor contagion, mimicry, and emotional contagion [6]. Notably, only empathic concern maintains a clear self–other distinction. Among the domains in which empathy manifests, the response to others’ pain—also known as *empathy for pain* [6]—has attracted considerable interest. Also referred to as *vicarious pain*, it reflects an internal simulation that evokes pain-like experiences upon observing someone else in pain [7]. Additionally, individual differences in empathy for pain have also been linked to bodily states and personal pain experiences. For example, chronic pain conditions are associated with altered neural processing of pain, including reduced activation of empathic networks [8,9,10]. Experiencing pain, particularly when it becomes chronic and maladaptive, can lead to alterations in physiological and psychological processes related to pain perception and pain-related behaviors [11,12,13]. Recent studies show that individuals who are in pain react less empathically to the pain of others [14]. Concerning empathy for pain, some theories of embodied cognition have tried to encompass the complexity [15] by proposing two complementary mechanisms: *experience sharing* and *mentalizing* [16]. Mentalizing refers to the cognitive ability to infer others’ mental states, such as beliefs or intentions, while experience sharing describes the affective tendency to resonate with or “feel” the emotions of others [16]. According to the shared-representation model of empathy for pain, perceiving and interpreting the pain of others is thought to rely on a simulation of experiencing pain and involves similar nodes of the “pain matrix” [17,18,19,20,21], including affective [21,22,23] and sensory areas [24]. This component, which is sensory-discriminative in nature, involves pain intensity and its somatic location, thereby activating primary and secondary somatosensory cortices (SI and SII), as well as the posterior insula [25,26,27].

The affective component implicates the anterior insula, anterior cingulate cortex, thalamus, and brain stem and correlates with the unpleasant nature of pain [28]. An alternative perspective, known as the Threat Value of Pain Hypothesis, suggests that the pain of others is not always an experience to be shared. Instead, it may function as a threat signal, capable of activating a fight-or-flight defensive response [29]. In line with this view, an elevated cortisol response to acute stress—reflecting the physiological mobilization needed to cope with a threat—has been associated with reduced empathy for others’ pain [30]. This may be due to the activation of the hypothalamic–pituitary–adrenal (HPA) axis, which reorganizes cognitive and emotional resources toward self-protection, thus impairing the capacity to perceive and share the suffering of others [18]. Under this interpretive model, individuals suffering from chronic pain may develop a heightened threat sensitivity, leading them to perceive pain as an additional stressor. This, in turn, may reduce the relevance attached to others’ pain and inhibit the empathic response [29,31].

Within this framework, Wulf and Tom (2023) [32] highlight how, following a spinal cord injury (SCI), the loss of descending control by supraspinal structures leads to dysregulation of the sympathetic nervous system, which tends to assume a hyperexcitable and reactive state to threatening stimuli. Such autonomic instability may interfere with the organism’s capacity to process others’ pain, ultimately compromising empathic engagement [32,33]. Therefore, spinal cord injuries are disruptive neurological events that severely affect the body, leading to the interruption of sensorimotor and autonomic pathways [34,35,36]. Recent research highlighted that SCI-related alterations extend beyond the expected network, involving most of the central nervous system, and go far beyond primary sensorimotor cortices [37]. However, neural activity is extremely adaptable, even over short time scales, and is modulated by changing conditions or different compensative strategies [38,39,40]. Building on this perspective, a few empirical studies have explored how empathy is expressed in people with SCI. Guadagni et al. (2019) found that patients with SCI are more likely to employ cognitive empathy strategies rather than engage in emotional simulation when interpreting affective cues [41]. Another study specifically examined how empathy for pain is expressed in individuals with SCI [42]. In that study, the authors explored the behavioral and physiological correlates of pain empathy in people with paraplegia following SCI, elicited by the observation of innocuous or painful stimuli applied to specific body parts. They found a reduced sensorimotor empathic response—as measured by facial muscle activity in the corrugator muscle—leading to the conclusion that SCI lesions may impair the sensorimotor components involved in empathy for pain. Although this topic is of considerable importance, clinical evidence remains limited, and the potential effects of chronic pain, exemplified by post-traumatic spinal cord injury pain, on vicarious pain responses are not well-documented. Here, compared to healthy controls, we explored (i) how pain intensity and unpleasantness, as well as complete deafferentation and de-efferentation, influence empathy-related responses; (ii) whether response times are comparable when observing painful versus non-painful stimuli from a third-person perspective (3PP); and (iii) whether, and how, empathic reactivity is modulated by acute short-term and long-term chronic pain.

To assess the empathic reaction to the pain of others, we recorded behavioral responses to an ad hoc devised classification task: manipulating noxious or non-noxious stimuli applied on a hand model in a third-person perspective. To clarify the relationship between SCI and empathic disposition, we administered the Interpersonal Reactivity Index [43] to participants with SCI and to healthy controls. To investigate the impact of both short-term and long-term subjective pain intensity and unpleasantness on sensitivity to others’ pain, a numeric rating scale (NRS) was used, in accordance with the Neuropathic Pain Scale (NPS). The behavioral mechanism of visual perspective taking in pain observation paradigms is a commonly documented approach [44,45,46]. Previous results have shown that seeing pain from different visual perspectives produces comparable neural activations, which allow sharing and understanding of the pain of others, even if the original viewpoint has changed [47]. Assuming that the awareness of self bodily-state is essential for identifying the same states in others [48], we expect that due to deafferentation and chronic pain, patients with SCI, as compared to healthy controls, would show weaker responses for painful stimuli together with blunted empathic responses. Finding different results between participants with SCI and healthy individuals, with no specific experience of chronic physical pain, would support the hypothesis that experiencing pain may produce a self-centered bias that eventually reduces pro-social behaviors like empathy. Because empathy is a potent predictor of helping behavior [6], studying its plasticity could be the first step toward the positive promotion of particular aspects of social interaction and rehabilitation [49]. From a clinical perspective, being aware of the effects of experiencing chronic pain on the perception of others’ pain might significantly improve pain assessment procedures [31]. Some studies have shown that awareness of bodily signals plays a prominent role in the experience of pain, affecting both sensory and linguistic responses to positive or negative expressions [50].

## 2. Materials and Methods

*Participants* In this case–control study, fifteen subjects with established SCI (13 men; age range 19–56 years) and 15 healthy control subjects without SCI (11 men; age range 25–48 years) were recruited at Santa Lucia Foundation Hospital in Rome, Italy. The neurological injury level of the patients (Table 1) was determined using the American Spinal Injury Association (ASIA) international standards for the classification of spinal cord injury. All participants with SCI in the study sample had a lower (thoracic) spinal cord injury that resulted in paraplegia with spared sensory and motor function of the upper segments of the body. Six were diagnosed with neuropathic pain, while the other nine suffered from other forms of chronic pain. All participants with SCI were recruited in the chronic injury phase (6 months post-injury), and none had reported a traumatic brain injury associated with the spinal cord injury. No participant presented signs of a psychiatric disorder, and none had a history of substance abuse. All subjects were right-handed, according to the 10-item version of the Edinburgh Handedness Inventory [51].

**Figure 1:**
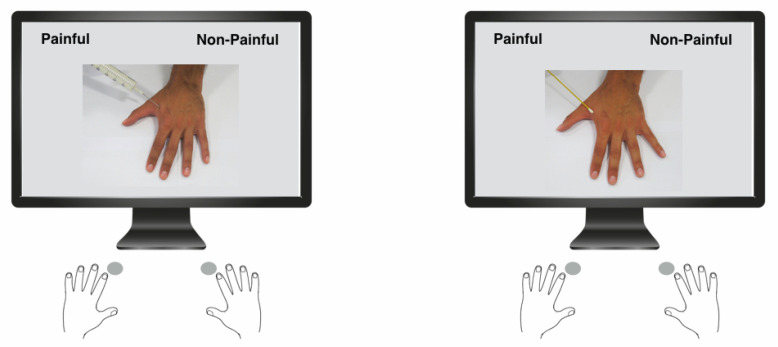
Experimental design. Examples of painful and non-painful stimuli are shown. Subjects pressed one of two buttons to classify the pictures as either “Painful” or “Non-Painful”.

*Behavioral Tasks* To investigate specific reactions to other people’s pain, we devised an ad hoc classification task that would reveal implicit reactions related to embodied pain. In each trial, a single image was shown on a screen, and participants were asked to categorize it as either painful or not painful. Category labels were displayed in the top left and right corners of the screen. Responses were given by pressing the corresponding left-hand (Q) or right-hand (P) key, with button assignment reversed and counterbalanced across participants. A 250 ms inter-trial interval followed each response. Participants were instructed to respond as quickly and accurately as possible, as we were interested in both accuracy ratings and reaction times. A red “X” appeared on the screen after incorrect responses [52]. Stimuli presentation and response were managed using E-Prime 2 software (Psychology Software Tools, Inc., Sharpsburg, PA, USA). The stimuli consisted of eight pain-related pictures depicting a hand with a syringe needle touching different points of its dorsal surface and eight non-pain-related pictures with a Q-tip touching the hand. All stimuli were presented from a third-person perspective (3PP), simulating pain or a gentle touch being inflicted on another person. Thus, the task involved two conditions (3PP-Painful vs. 3PP-Not Painful), each comprising eight unique stimuli. Each image was presented twice for a total of 32 trials (16 painful and 16 non-painful), randomized across participants to prevent expectancy effects. All pictures were taken from a male model (30 years old) who did not take part in the experiment. Given that all SCI participants had lower spinal cord injuries, using pictures of an upper body segment allowed the investigation of resonant pain processing in body parts with normal mobility and sensitivity. A schematic representation of stimuli and procedure is displayed in Figure 1.

Evaluation Instruments Participants also completed the Interpersonal Reactivity Index (IRI) [43], a self-report measure of empathic dispositions, comprising 28 mixed positive and negative statements. Using a five-point Likert scale (ranging from 1 [“Does not describe me very well”] to 5 [“Does describe me very well”]), participants rated each statement. The questionnaire consisted of four subscales, each containing seven items: Perspective Taking (PT, the ability to adopt someone else’s point of view in order to better understand his or her behavior), Empathic Concern (EC, the propensity to experience sympathy and compassion for others), Fantasy Scale (FS, the tendency to shift oneself into imaginary situations), and Personal Distress (PD, the predisposition to feel distress and anxiety in response to others’ misfortunes). The IRI remains a widely used standard tool for measuring empathy, where higher subscale scores reflect stronger empathic abilities. The IRI demonstrated good internal consistency across all subscales, with test–retest reliability correlations ranging from 0.61 to 0.81 [1].

To introduce a subjective evaluation of pain for every patient, both “pain intensity” and “pain unpleasantness” were also assessed on a numeric rating scale (NRS) from 0 (“no pain”) to 10 (“worst pain imaginable”) in accordance with the Neuropathic Pain Scale (NPS) [53,54]. Considering the time periods of one week and eight months prior to the study ensured that the entire period of reference was shorter than the time since injury (minimum for all our participants with SCI being 260 days). This approach allowed us to investigate the influence of acute short-term and long-term subjective pain on the processing of others’ pain.

### 2.1. Procedure

The study was approved by the ethics committee of the Santa Lucia Foundation and conducted in accordance with the Declaration of Helsinki. Participants were first informed about the experimental procedure and then asked to provide written informed consent for their participation. They were then asked to sit at a distance of approximately 50 cm from a 17-inch screen (resolution: 1024 × 768 pixels) to complete the categorization task. After completing the task, participants were asked to fill out the IRI and to answer pain-related questions.

### 2.2. Data Analysis

Each subject’s reaction times (RTs) on the categorization task were averaged for painful and non-painful trials. Trials with incorrect responses (3.75% on average) were discarded from the analysis. The accuracy rate was calculated as the percentage of correct responses of all the valid trials. As the pictures were very clear and easy to classify, all subjects were extremely accurate in performing the task, with a very high accuracy rate (96% on average) for both the conditions (3PP-Painful: 97%; 3PP-Not Painful: 95%; t_(28)_ = 0.91, *p* = 0.28). Therefore, the performance was further analyzed solely in terms of reaction times. We conducted an ANOVA on RTs with the type of stimuli (painful vs. not painful) as the within-subject variable and group (SCI participants vs. HC controls) as the between-subject variable. All pair-wise comparisons were performed using Fisher’s post hoc test. A significance threshold of *p* < 0.05 was set for all statistical analyses. The data are reported as the mean ± standard error of the mean (SEM). The global score from the IRI was computed by summing the single scores obtained in the different subscales. This score was then analyzed with the Mann–Whitney *U* test. To further investigate empathic dispositions in our sample, we also analyzed the individual subscales separately. Moreover, non-parametric correlation tests (Spearman’s rank correlation coefficient) were run for the scores on the IRI, RTs, SCI participants’ subjective ratings of pain “intensity” and “unpleasantness,” age, time since injury, and lesion level.

## 3. Results

**Figure 2:**
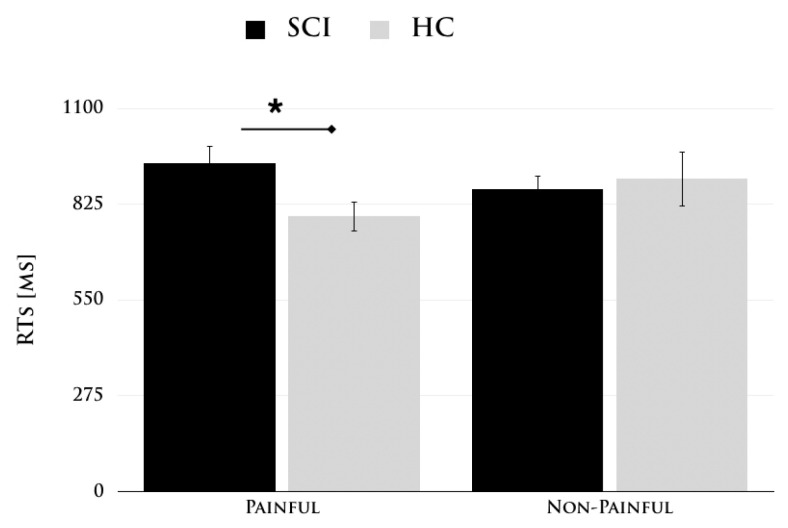
Results from the classification task. The RTs obtained from the two groups of participants (SCI: participants with SCI, HC: healthy controls) in the empathy for pain task are shown. The error bars indicate standard errors of the mean (SEM). The asterisks (*) indicate significant results.

*Empathy for pain* The ANOVA run on the RTs for the categorization of painful and not painful stimuli revealed no main effect by group (F_(1,1)_ = 0.87; *p* = 0.35; η_p_^2^ = 0.03) or type of stimuli (F_(1,1)_ = 0.15; *p* = 0.69; η_p_^2^ = 0.005). Crucially, an effect of interaction was found (F_(1,28)_ = 4.59; *p* = 0.04; η_p_^2^ = 0.14): participants with SCI (942.11 ms ± 51.08) were significantly slower than healthy controls (789.84 ms ± 41.92) in classifying painful stimuli, suggesting that the avoidance response to the vision of another’s pain was less pronounced in these participants. As expected, no such difference was found for non-painful stimuli (SCI = 867.64 ms ± 40.18; H = 897.65 ms ± 78.78; all p_s_ > 0.20). Data are reported in Figure 2.

**Figure 3:**
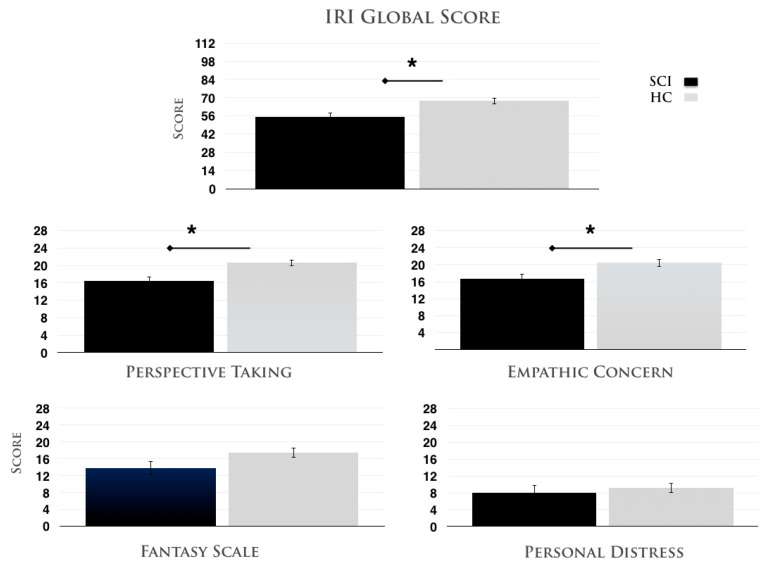
Interpersonal Reactivity Index scores. The global score obtained from the two groups of participants (SCI: participants with SCI, HC: healthy controls) in the IRI is shown in the top panel. The subjective ratings of the four different subscales are shown in the lower panels. The error bars indicate standard errors of the mean (SEMs). The asterisks (*) indicate significant results.

*Dispositional empathy* Concerning the global IRI score, the median scores in the group of participants with SCI and the group of healthy controls were 173.5 and 291, respectively, and the distributions in the two groups differed significantly (Mann–Whitney *U* = 53.5, *n*1 = *n*2 = 15, *p* < 0.01 two-tailed), with SCI participants generally less empathic (mean SCI = 55.06 ± 3.64) than healthy controls (mean HC = 67.6 ± 2.46). We also found a specific difference according to the different subscales. Specifically, in Perspective Taking (Mann–Whitney *U* = 37, *p* < 0.001 two-tailed) and Empathic Concern (Mann–Whitney *U* = 56.5, *p* < 0.02 two-tailed), participants with SCI (PT = 16.4 ± 0.97, EC = 16.73 ± 1.07) obtained significantly lower scores than did healthy controls (PT = 20.6 ± 0.67, EC = 20.4 ± 0.83). On the other hand, in the Personal Distress subscale (Mann–Whitney *U* = 86, *p* < 0.28 two-tailed; SCI = 8.06 ± 1.71; H = 9.13 ± 1.13) and in the Fantasy Scale (Mann–Whitney *U* = 71, *p* < 0.09 two-tailed; SCI = 13.86 ± 1.58; H = 17.46 ± 1.21), no differences were present, even if a trend was visible in the latter. These different results for the four subscales exclude the possibility of general answering biases. All data are reported in Figure 3.

### The Impact of Pain on Empathy

**Figure 4:**
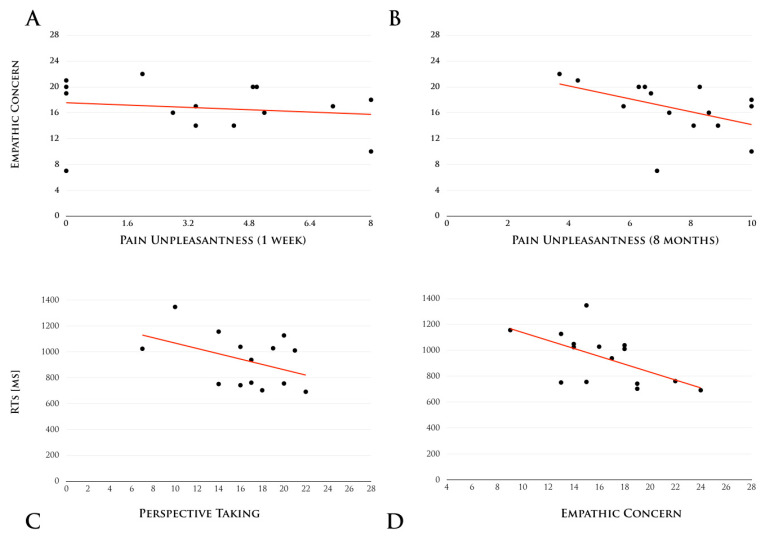
The impact of pain on empathy. Scatter plots showing the SCI participants’ subjective ratings on EC as a function of pain unpleasantness, as assessed at the time of the study and eight months prior it, are shown in panel (**A**,**B**); scatter plots showing the mean RTs for painful stimuli as a function of Perspective Taking and Empathic Concern for participants with SCI are shown in panels (**C**,**D**), respectively.

A significant inverse correlation was also found between the scores on the PT subscale and RTs for the categorization of painful stimuli in SCI participants (r = −0.63; t = −2.90; *p* = 0.01), meaning that the less SCI participants were able to understand another person’s point of view, the more slowly they reacted to his or her pain (see Figure 4). No such correlation was found for non-painful stimuli (r = −0.39; t = −1.54; *p* = 0.15). Additionally, we observed a significant inverse correlation between subjective ratings of pain “unpleasantness” in participants with SCI and the EC subscale of the IRI. Specifically, the higher the SCI participants rated chronic pain as unpleasant (mean = 6.1, r = −0.62; t = −2.83; *p* = 0.01), the more likely they were to have lower scores in the EC (Figure 2). Interestingly, this effect is explained from the “unpleasantness” of the chronic pain (as assessed at eight months prior to the study) and not in the last week (r = −0.29; *p* = 0.29), suggesting that experiencing chronic pain reduces the capacity for empathic concern toward others. No other correlation was found to be significant in the group of SCI participants, and there were no correlations found in the healthy controls.

## 4. Discussion

Pain appears to be intimately linked with both physical [55] and emotional states [10]. Here, we demonstrate that self-pain modulates empathy in participants with SCI. In particular, we show that the condition of chronic pain makes an individual less inclined to react in a pro-social manner. Sensorimotor body–brain disconnection in SCI individuals might affect sensorimotor dimensions connected to empathy for pain. To assess this hypothesis, we administered the IRI and a behavioral classification task involving painful and non-painful stimuli presented from a third-person perspective to healthy participants and individuals with complete thoracic spinal cord lesions [43]. Compared to healthy controls, individuals with SCI scored significantly lower on the Perspective Taking and Empathic Concern subscales of the IRI. Neural changes in SCI and neuropathic pain may be partially related to low empathy scores. Alterations in somatosensory processing are often reported in individuals with SCI [37]. Several studies suggest an engagement of the somatosensory cortices for state and trait empathy [56,57,58,59,60]. Recent research identified structural differences associated with myeloarchitectural integrity in the somatosensory cortices for the cognitive component of empathy [26,61]. The tendency to adopt the psychological point of view of others and the capacity to understand their emotions and respond properly represent fundamental features of empathy in everyday life [46]. Thus, the low empathy scores in participants with SCI is naturally of great impact for their social interactions and can lead to important consequences in their daily lives, as both cognitive and emotional aspects of empathy have a direct positive effect on the support of others [62] and emotions [63]. These findings contrast with those of Guadagni et al. (2019) [41], who, in examining both cognitive and emotional empathy in SCI patients, found the only statistically significant difference in the IRI was that SCI patients reported higher scores on the Perspective Taking subscale [41]. However, that study did not consider chronic self-pain as a possible variable impacting empathy. Moreover, the patients exhibited sensory deafferentation and motor de-efferentation as a consequence of high cervical and incomplete lesions (AIS grade B and C). Discrepancies between the results of the two studies suggest that pain directly resulting from SCI lesions could potentially impact sensorimotor, cognitive, and affective empathy and chronic self-pain can eventually reduce one’s responsiveness to the pain of others. Therefore, we found that greater chronic pain unpleasantness was associated with lower empathic concern in participants with SCI, suggesting that the experience of chronic pain may diminish the empathic dispositions. According to the IRI scores, the way participants with SCI responded to others in pain differed from both healthy controls and their own reactions to stimuli that did not involve pain. It is important to note that cortical pain processing also provides the resources for adaptive escaping or avoidance reactions to pain [64]. Our results suggest that self-pain can eventually reduce one’s responsiveness to the pain of others [31]. The longer reaction times (RTs) of SCI participants in response to painful stimuli presented from a third-person perspective (3PP) may reflect a diminished empathic reaction toward others’ suffering. Additionally, pain’s multifaceted effect on behavior involves a strong motor component, directly recruiting brain regions connected to voluntary action [65]. In particular, specific motor-related areas are crucially involved in the regulation of behavioral reactions to pain [64]. Moreover, observing another’s pain modulates the activation of these motor-related regions and the neural activity elicited by a concurrent nociceptive stimulus [66]. Studies on empathy for pain indicate that people who see or imagine others in pain tend to empathically share what others feel at both the behavioral and neural levels, a process mediated by the Mirror Neuron System (MNS) [10,45,67]. In particular, some studies have reported, the MNS’s role in empathy, considering the activation of cortical regions involved in the direct experience of pain (i.e., ACC and insula) is associated with activation of central regions of the sensorimotor areas during the observation of others’ pain [10]. Thus, at the neurofunctional level, in empathy for pain there is a partial overlap between the areas activated during the direct experience of pain and the observation of others’ pain. However, this overlap is accompanied by a functional segregation within the pain matrix, reflecting the need to maintain a clear distinction between self and other. Indeed, empathic capacity involves not only affective sharing of the other’s emotional state, but also the ability to distinguish between self and other at the representational and bodily levels. This distinction is crucial to avoid overidentification and ensure an accurate understanding of another’s emotional state [3]. An impairment of this distinction can foster personal distress and hinder prosocial behaviors [3]. Experimental evidence indicates that sensorimotor areas, including the premotor and somatosensory cortices, contribute to empathic pain perception by supporting embodied simulation processes [68]. 

The perceptual-motor codes and the integrity of body signals thus appear essential for effective empathic processing. In support of this claim, studies of individuals with chronic pain have shown reduced activation of somatosensory cortices during the observation of others’ pain, indicating a possible weakening of the embodied component of empathy [69]. Moreover, viewing another’s pain modulates the activation of motor-related areas and the neural activity arising from a concurrent nociceptive stimulus [66]. In the case of participants with SCI, this activation and, therefore, the shared-representation model of pain empathy, ref. [48] might not be applicable due to the cortical reorganization occurring in sensorimotor areas [70]. Assuming that empathy may shape sensorimotor resonance [71], it can be hypothesized that these findings could be explained by an altered direct sensorimotor mirroring mechanism driven by chronic pain and the brain–body disconnection after SCI. While our results are consistent with those of the aforementioned literature, the current study cannot exclude the possibility that other mechanisms might cause the SCI participants’ altered responses to the pain of others. A recent study [69] showed that greater interoceptive sensitivity shapes both cognitive and emotional empathy, allowing one to feel more compassion for others in pain. Participants with SCI have shown reduced interoceptive awareness [72,73,74,75]. Thus, interoceptive sensitivity might be responsible for the lack of empathy for pain that was revealed in our results [76]. On the other hand, SCI participants’ reaction to non-painful stimuli was comparable to that of healthy participants. Gentle touch perception, and its related pleasant sensation, is associated with a modulation of the posterior insular activity [77], which might be completely spared in our sample of participants with SCI. A study [78] revealed that pleasant touch was also associated with higher medial orbitofrontal cortex activation. Therefore, two distinct neural networks may be recruited to process the positive and negative stimulation of others [78]. Thus, we believe that the dissociation of these brain networks should be a focus of study in future research regarding empathy for pain [79]. Although our findings support an embodied cognition approach, many other factors are likely to modulate the development of empathy for pain [80]. Social relationships between the parts and affective links [23], perceived similarity [81], social membership [52,82], and racial origin [19] have already been investigated. Despite its pro-social function, empathy for pain is likely to fail if the sufferer is regarded as socially distant. Although people tend to consider themselves as pro-social individuals, there are many circumstances in which they think, feel, and act largely in an egotistical manner, often failing to empathize to the same extent with out-group members [80,83]. It has been repeatedly demonstrated that people are less prone to help out-group members [84] and less likely to value their lives [85]. Furthermore, in these cases, it seems possible that experiencing pain causes individuals to react less empathically to the pain of others. Therefore, the findings from the present study may provide important insight to facilitate further development of therapies to restore empathic concerns, which may have a positive social impact. Empathy plays a crucial role in the inter-individual sharing of emotions, feelings, and beliefs that characterize human social interactions. For individuals living with chronic pain, alterations in empathic processes can significantly influence their relationships with healthcare professionals, the support they receive from caregivers and family members, their social functioning, and ultimately, their overall quality of life [86]. Although psychiatric disorders—including depression—were exclusion criteria in our sample, chronic pain is frequently associated with mood disorders, as it is considered a chronic stressor [87]. In fact, individuals with depression often exhibit diminished emotional empathy [88,89] and reduced capacity for both affective and cognitive theory of mind, potentially leading to more self-focused or blunted empathic reactions [90,91]. Depression is also a common comorbidity after SCI, with a mean prevalence estimate of around 22% [92]. Although our participants did not meet the criteria for such diagnoses, these factors may still be relevant to consider when interpreting our findings in a broader context, as they might contribute to altered empathic processing in the wider SCI population.

## Figures and Tables

**Table 1:** Clinical data of the participants with SCI. Reported: time since injury (days), clinical neurological level and height of the lesion (T = thoracic), neurological and functional levels of the injury as determined by the American Impairment Scale (AIS), and subjective ratings on pain “intensity” and “unpleasantness.”.

Case	Days Since Injury	Lesion Level	AIS Grade	Pain Intensity	Pain Unpleasentness	Pain Intensity	Pain Unpleasentness
				1 Week		8 Months	
**P1**	699	T10	A	4.2	5.2	8.5	8.6
**P2**	840	T3	A	4.5	8	8.6	10
**P3**	360	T10	A	0	0	6.1	6.9
**P4**	260	T12	A	2	2.8	7.4	7.3
**P5**	1095	T9	A	0	0	2.2	4.3
**P6**	3850	T5	A	2	2	2.6	3.7
**P7**	6040	T5	A	3.2	3.4	5.5	5.8
**P8**	2057	T12	A	0	0	7.5	6.7
**P9**	2520	T3	A	3	4.9	6.5	8.3
**P10**	2305	T8	A	2.4	4.4	5.2	8.1
**P11**	2580	T4	A	5.7	7	10	10
**P12**	750	T12	A	2	5	4.6	6.5
**P13**	360	T7	A	0	0	5.6	6.3
**P14**	5745	T6	A	2.8	3.4	7.4	8.9
**P15**	435	T10	A	6.2	8	10	10

## Data Availability

Data that support the findings of this study are available from the corresponding author upon reasonable request.
